# A more just union: Euro‐dividend or reinsurance?

**DOI:** 10.1111/ejop.12731

**Published:** 2021-10-22

**Authors:** Andrea Sangiovanni

**Affiliations:** ^1^ Department of Philosophy King's College London UK

## Abstract

What principles of social justice apply to the European Union? This paper has three parts that together challenge Philippe Van Parijs's most recent proposals for how to think about justice in Europe. For Van Parijs, the EU represents an opportunity to begin globalizing a universal basic income that should, in time, be extended to the whole planet. In the first part of this paper, I recapitulate Van Parijs's main line of argument. In the second, I query the philosophical grounds for Van Parijs's argument that the EU must become a fully‐fledged federal transfer union—a union, that is, that aims to attain an egalitarian distribution across all European citizens taken as individuals (rather than as members of nation‐states). In the third, I challenge whether a European Basic Income—the euro‐dividend—would work, as a policy, in quite the way envisaged by Van Parijs. I then conclude with some implications for how to think about justice for the EU.

## 1 INTRODUCTION

In 1998, Philippe Van Parijs and John Rawls exchanged letters about Rawls's soon‐to‐be‐published *Law of Peoples*. The European Union (EU) was at the heart of their debate. In the *Law of Peoples*, Rawls rejected the cosmopolitan idea that his two principles of justice apply to international relations. Instead, he defended the idea that peoples only owed stringent duties of assistance to what he called “burdened societies” (societies that struggled, that is, to sustain stable polities sufficient for economic development beyond a bare minimum). As many of his students and colleagues were surprised to find, more demanding principles of international distributive justice beyond a human rights minimum were not in the cards. But what about the EU? The EU is a union of peoples that is not a state but not an international organization either. What principles of distributive justice ought to apply there? Van Parijs pressed Rawls to clarify whether the “emerging political entity [the EU] will (and should) never be more than a conglomerate of ethnoi‐demoi, between which only assistance is required on grounds of justice, or whether it can constitute a poly‐ethnic demos to which a more demanding conception of distributive justice can conceivably apply.” (Van Parijs & Rawls, 2003)

The question was a good one for two main reasons. First, it challenged Rawls to clarify how much and what kind of institutional cooperation is required to trigger principles of distributive justice. Forcing Rawls to consider a case in which his highly idealized, some would say outdated, for example (Buchanan, 2000), model of international relations self‐evidently failed would push him to confront issues that were only mentioned in passing in the *Law of Peoples*. Why, for example, should we assume that only peoples have basic structures? Why not unions of peoples, each of which retains its nationhood? And, indeed, what about the broader institutional infrastructure of globalization—the formal and informal institutions and organizations that govern areas from satellite communications and security cooperation to trade, global health, and the environment? If these structures are profound and pervasive from the start, often leave states with no option but to participate, and set up extensive patterns of cooperation and reciprocity, why *should not* principles more demanding than assistance but less demanding than the Two Principles apply?

Second, Van Parijs was beginning to wonder whether the EU needed its own theory of justice (Van Parijs, 1997, 2000). The question had become urgent during debates on the Constitutional Treaty, which was intended to replace the bundle of treaties governing the EU with a single text, and, more optimistically still, to set the EU on a more federal path. Most of the normative and theoretical EU literature at the time was focused on the so‐called “democratic deficit.” Almost nothing had been written, by contrast, on principles of distributive justice (“solidarity”) at the EU level.^1^ Asking Rawls, the most influential political philosopher of the twentieth century, what he thought about the issue might serve to illuminate and help in shaping the debate.

But Rawls's answer was, to my mind, evasive. Rawls did not confront the EU as it was. Instead, he asked what we should think if “Belgium and the Netherlands, or the two together with France and Germany, decide they want to join and form a single society, or a single federal union.”^2^ It is of course true that, *if* these countries explicitly agreed to join and form “a single society, or a single federal union,” principles of liberal justice would now apply to their union (just as in the case of multination peoples). But even if we knew what Rawls meant by a “single society, or single federal union” (a state?), no member state had explicitly agreed to join such a single society.^3^ So the question remained: Do principles more demanding than assistance but less demanding than the full extent of liberal justice apply to the EU, or not? If so, what are they, and how would we go about constructing them?

There are resources that Rawls could have drawn on to answer the question. In the *Law of Peoples*, there is a section on “cooperative organizations,” by which Rawls intended to refer to organizations like the World Bank, the WTO, and the UN. He writes that such organizations should be governed by suitable principles of background fairness and reciprocity and that these principles should be decided by peoples choosing behind a veil of ignorance (such that they would not know “whether [their] economy is large or small”). Rawls does not say much more about what kinds of principles peoples behind such a veil of ignorance would choose. Instead, he writes the following: “Should cooperative organizations have unjustified distributive effects between peoples, these would have to be corrected, and taken into account by the duty of assistance.” (Rawls, 1999, p. 43) But this is, once again, equivocal. Does Rawls mean that distributive effects between peoples are unjustified *only* if they fail to satisfy the duty of assistance, or does he mean that failing to satisfy the duty of assistance is only *one* way in which they could be unjustified? We are left wondering what other principles there might be, and how one should go about determining them.

The questions raised by Van Parijs in 1998 have only become more pressing. Two years later, then‐Advocate General Miguel Maduro bemoaned the state of the debate on the Constitutional Treaty and called for a deeper reflection on the “criterion of distributive justice” that should guide European reform. “Without such a debate,” he wrote, “there can be no true social contract capable of legitimizing the emerging European polity and the consequences would be either a return to a less advanced form of integration . . . or, if the current model continues to be stretched, a crisis of social legitimacy which may manifest itself in increased national challenges to European policies (whose redistributive effects are not understood and accepted).” (Maduro, 2000, p. 347)

Maduro's challenge was prescient. In 2005, the Constitutional Treaty was rejected by the Dutch and French in a series of referenda. It was widely accepted that one of the main reasons was the growing perception that the EU tended to undermine the welfare state by promoting market integration while not doing much by way of market correction. Since 2008—in the long shadows cast by the eurozone, refugee, Brexit, and COVID‐19 crises—the distributional consequences of European integration have grown in salience and become dangerously destabilizing. There is no way one can begin or end a debate on the EU today without addressing what kind of solidarity Europeans owe one another in virtue of their shared commitments to peace and prosperity.

This article has three parts designed to challenge Van Parijs's most recent proposals for how to think about justice in Europe. For Van Parijs, the EU represents an opportunity to begin globalizing a universal basic income (UBI) that should, in time, be extended to the whole planet. In the first part of this article, I recapitulate Van Parijs's main line of argument. I will only address his arguments regarding socioeconomic justice. Van Parijs has also defended views on linguistic justice and how to reorganize representative institutions at the EU level. I leave these aside in what follows. In the second, I will query the philosophical grounds for Van Parijs's argument that the EU must become a fully fledged federal transfer union—a union, that is, that aims to attain an egalitarian distribution across all European citizens taken as individuals (rather than as members of nation‐states) and demotes member states to tools necessary for achieving this end. In the third, I challenge whether a European Basic Income—the euro‐dividend—would work, as a policy, in quite the way envisaged by Van Parijs. I then conclude with some implications for how to think about justice for the EU. The aim, in a way, is to continue the debate that Van Parijs started with Rawls, picking up where they left off.

## 2 JUSTIFYING THE EURO‐DIVIDEND

Van Parijs's argument concludes that the EU should adopt, as a temporary measure foreshadowing more thorough‐going reform, an unconditional basic income of about €200 a month (adjusted for cost of living) for each EU citizen and resident. Funding such a modest income would require extracting 8% of EU GDP in taxes; Van Parijs suggests that this could best be accomplished through a VAT increase of up to 19% in each member state, which would then go directly into EU coffers (the EU already gathers a portion of its own resources through a commonly defined VAT base).^4^ In this and the next section, I discuss the philosophical argument Van Parijs uses to justify this conclusion in “Just Europe.” In the next, I discuss the merits of the proposal as a policy.

The most powerful argument for a UBI appeals to the idea that we all benefit from a common inheritance that none of us is individually responsible for producing. The jobs that are available, the structure of the market that largely determines what counts as a talent, the education we have received to acquire those talents, and the pattern of preferences, skills, and capital that determines prices and wages are all a result of the existence of a background stock of cultural, political, economic, and social capital stored and reproduced primarily as institutional knowledge passed from one generation to the next. Herbert Simon, for example, estimates that 90% of our earned income is generated not by isolable acts of talent or effort but by the positive externalities of living in a well‐functioning state with a wide and reliable provision of collective goods (Simon, 2000). If this is true, then a flat tax of up to 90% would seem, in principle, fully justifiable—a way of returning that wealth back, as Simon writes, to “its rightful owners.”

But this covers only the supply side. What then to do with that revenue? Van Parijs's suggestion is that it ought to be redistributed directly to citizens as a UBI—an income that represents everyone's stake in the capital that we are all responsible, collectively but not individually, for producing and reproducing. It is *universal* because unconditional: Every individual, whatever their propensity to work, age, marital status, or their level of income or wealth, should receive it. It is *basic* because it should be high enough to give everyone the freedom to say “no” to bad, exploitative jobs, and “yes” to whatever they want to do, whether raise a family, paint, or seek further training. A UBI pitched at the maximum sustainable level, in short, provides the most promising means for expanding (or, more precisely, leximinning) what Van Parijs calls “real freedom.”

In the previous paragraphs, I referred to “we” and to “every individual” but over whom, exactly, do those terms range? Do “we” and “every individual” include all human beings, or to citizens and residents of states? In previous work, Van Parijs offered mainly functional arguments for “scaling up” the UBI, arguing that merely domestic UBIs would be unsustainable in an era of footloose capital and selective migration(see, for example, (Van Parijs, 1995, pp. 226–234)).^5^ In “Just Europe,” he adds a moral argument.

The argument depends on a weakened version of Thomas Nagel's two conditions for triggering demands of egalitarian justice (Nagel, 2005). For Nagel, egalitarian justice only applies when and because (a) societal norms are imposed coercively, and (b) those subject to the norms are both their authors and addressees. Nagel believes that these conditions only apply at the state level. International treaties, regimes, and institutions (including, he claims, the EU) are voluntary: States are not forced to join or participate. They also do not claim to “speak in the name” of individuals but of states, who are the agents expected to comply with them. So if citizens have a complaint with respect to international arrangements (including their distributional consequences), Nagel argues, the appropriate arena to seek justification and redress is the national government responsible for entering the arrangement in the first place.

Van Parijs accepts the premise that coercion is necessary but believes that the authorship condition is too strong. What is required to trigger egalitarian justice is the existence not of a fully fledged *demos* but of a “justificatory community”—a community constituted by channels of communication and deliberation such that people can demand justifications and expect answers to them. Because, by now, states and their citizens cannot but comply with wide swathes of EU norms, and because of the “expansion of traveling and transnational media, the spreading of lingua francas and the Internet, the widening and thickening of international organizations and of the global civil society,” (Van Parijs, 2019, p. 16), the conditions for egalitarian justice, and hence Van Parijs's UBI, are satisfied at the EU level. They are also met, Van Parijs avers, at the global level, especially once one considers the expansive network of norms and institutions that quickens and deepens the distributional effects of globalization on the population of all states. As Van Parijs writes,

Those who have some say over the patchwork of coercive rules that currently form the global basic structure owe a justification for these rules to those expected to comply with them: not just motives to obey them, such as the sheer fear of sanctions, but reasons to accept them as free and equal human beings. (Van Parijs, 2019, p. 16)^6^

In turn, the only justifiable reasons, Van Parijs argues, are egalitarian ones whose aim is to mitigate the arbitrariness of natural contingency and social fortune. And since a UBI at the maximal sustainable level is the best implementation of egalitarianism, the UBI be should scaled up, first to the EU, and then the globe as a whole.

## 3 THE IRRELEVANCE OF COERCION AND JUSTIFICATORY COMMUNITY

Van Parijs's argument for globalist/EU‐wide principles of justice inherits the same problems as coercion‐based approaches such as Nagel's. I will mention three of them.

First, it is not clear what work “justificatory community” does in the argument. In Nagel, it is essential that the people expected to comply with the norms are also, indirectly, responsible for imposing them on others. Because their agency is directly implicated in this imposition, they can demand a special justification that someone who is merely subject to coercion cannot. But once the “authorship” condition is dropped, it is no longer clear what normative role the mere existence of “justificatory community” is meant to play.^7^ At one point, Van Parijs writes that justificatory community is necessary for egalitarianism to “make sense.” (Van Parijs, 2019, pp. 16–17). If he means that coercion is sufficient, on its own, to trigger egalitarianism, and egalitarianism, in turn, requires the creation and maintenance of a justificatory community, then “justificatory community” is not a normative precondition for the application of principles of egalitarianism, but a further consequence of it. This would be fine, but it is then misleading why Van Parijs mentions it as an individually necessary and jointly sufficient condition for egalitarianism to apply in the first place. Coercion would be enough on its own to trigger both egalitarianism and the justificatory community thatought to accompany it.^8^

If, instead, he means that justificatory community really is an individually necessary and jointly sufficient condition for egalitarianism to apply in the first place, then I am unsure why. Suppose two groups, A and B, coercively maintain a UBI paid out, equally, to all of them, and suppose further that they enjoy justificatory community. Now suppose that A decides to close off whatever institutional channels Van Parijs imagines are necessary for justificatory community and then goes on to maintain the set of coercive structures that binds members of B, but no longer honors the pre‐existing egalitarian commitment to a UBI. Would the members of B now lack a claim in justice to a UBI as a result? If justificatory community is truly necessary, then it seems that they would, despite the fact that members of A continue to expect their (nonvoluntary) compliance. This looks implausible.

Second, even leaving the prior problem aside, coercion does not seem necessary for egalitarian justice to apply. Consider this case.

Imagine an internally just state. Let us now suppose that all local means of law enforcement—police, army, and any potential replacements—are temporarily disarmed and disabled by a terrorist attack. Suppose further that this condition continues for several years. Crime rates increase, compliance with the laws decreases, but society does not dissolve at a stroke into a war of all against all. Citizens generally feel a sense of solidarity in the wake of the attack, and a desire to maintain public order and decency despite the private advantages they could gain through disobedience and noncompliance; this sense of solidarity is common knowledge and sufficient to provide assurance that people will (generally) continue to comply with the law. The laws still earn most people's respect: the state continues to provide the services it always has; the legislature meets regularly; laws are debated and passed; contracts and wills drawn up; property transferred in accordance with law; disputes settled through legal arbitration, and so on. (Sangiovanni, 2007)

If coercion is a necessary condition, then egalitarian obligations would no longer apply among the citizens and residents of the post‐attack society. This, too, looks implausible: Were a group of right‐wing citizens to mobilize support for a nightwatchman state, the worst off would still have reason to complain.

One might object that the example is unrealistic. But why is that relevant? The point of the example is not to describe an empirical possibility, but to describe a case in which we hold constant everything but the presence of coercion. The hypothetical, furthermore, is not as remote as one might at first think. Most compliance with current legal systems occurs not because people are responding to the threat of being punished, but because, for a variety of reasons, they find the norms either sufficiently authoritative or find that noncompliance would be wrong for other reasons (e.g., because, say, murder or stealing is wrong, or out of a sense of fair play [when contemplating, say, cheating on their taxes], or simply out of habit). See, for example, Tyler (2006). No legal system could survive if individuals sought to disobey whenever they could get away with it for some personal gain; no amount of police or surveillance would be sufficient to prevent breakdown.

Alternatively, one might worry that the example only works because there is a more subtle form of coercion at work—a form of coercion that is sufficient to generate the very same demands of justice as more typical forms of state coercion (viz. those involving the threat of punishment and police enforcement). Isn't there, for example, still the threat of exclusion from the protection of the legal system, and hence the threat of being returned to the state of nature? This (implicit) threat, one might believe, still infringes the autonomy of those in the society, and so still must be justified in the same way.

But the example can be clarified in a way that makes it evident that no such threat is required for the example to retain its force. Suppose that it is well known that, should anyone stop paying taxes, nothing will happen to them.^9^ They would still have all the rights and privileges of citizens; the society has given up on the attempt to punish or penalize non‐compliers (though it still expects and calls for their compliance as a matter of law). And imagine that everyone continues to comply with the law out of a sense of solidarity and fair play, and that there is common knowledge of this fact. I do not see why the mere possibility of violating the law without suffering any sanction ought to change our judgment about the justice of the egalitarian scheme. Again, everything is as before: laws passed, taxes collected and resources redistributed, jobs completed, and so on. Why should the mere fact that people are no longer coerced make any difference to our claim on a fair share of the social product?

The last reason to reject the normative justification Van Parijs gives for scaling up his principles of justice is that coercion and justificatory community aren't even *sufficient*, either jointly or individually, for triggering egalitarianism. The fact that A *coerces* B to X undoubtedly raises the bar for what it would take to justify getting B to do X (compared with, for example, offering them an incentive). But the fact of coercion does nothing to set the terms in which the justification must occur. Depending on what X is and how the coercion takes place very different kinds of justification will be required.^10^ Suppose, for example, that A coerces B to obtain usage of his car, which he will use to save 10 people from drowning. The coercion seems justified, but it would be odd to conclude that, henceforth and as a result, A also requires obligations to maintain distributive egalitarianism between them. (Note further that the presence of “justificatory community” between A and B does not seem to change anything either way.) The *content* of the relevant justification depends on the target and context of coercion, rather than merely on the fact of coercion itself.

Van Parijs's conditions seem, I conclude, to need supplementation. Which conditions should we add? One candidate might be to require in addition that the object of the coercion, X, have profound and pervasive effects on B. But the same problem recurs. Different kinds of profound and pervasive effects will occasion different kinds of justification. Why should the justification for coercively getting someone into drug rehab, say, have the same content as securing, via coercion, a comprehensive set of public goods for an entire population? There is nothing in the idea of a profound and pervasive effect in itself that tells us what kinds of justifications are required just as there is nothing in the idea of a profound and pervasive effect that tells us only distributive egalitarianism can justify it. We need something more about the context before we can draw our conclusion. We also need a bridging principle that takes us from empirical facts about the domain in which the coercion/effect operates to the moral standards that should govern it.

The fact that neither coercion, profundity, pervasiveness, nor justificatory community is sufficient, and that, in all the cases discussed, we need something more about the domain in which each of these operates suggests that *what* a particular set of institutions does is more important in identifying which principles of justice apply than *how* it does it. This is especially evident in the terrorist attack case. What might explain, in that case, why principles of egalitarianism still apply? I have argued elsewhere that the most convincing answer appeals to the fact that the citizens and residents of the state continue to provide one another with a central class of collective goods necessary for a flourishing life. From this point of view, assurance secured through coercion looks like it is itself one of those collective goods, whose function is to provide assurance of compliance with law. Once that function is taken over by norms of fair play and solidarity, there is nothing left normatively or empirically for coercion to do. Above I also suggested that there must be a bridging principle that takes us from facts about a particular situation to egalitarian standards. In our case, that principle is reciprocity: citizens and residents owe one another a fair return for the mutual provision of a central class of collective goods.

If we further add the very same idea that Van Parijs uses to justify the supply side of the UBI, namely, that we are the happy beneficiaries of an institutional inheritance, secured through the provision of publicly backed collective goods, then we can also argue that it would be grasping to insist, based on reciprocity, for a return that is equivalent to the entire marginal product of our labor. We mostly owe what we can get from our talents and abilities on the market—recall Simon's 90%—to the positive externalities of living in a society with a well‐developed institutional order. We therefore owe all those who have contributed to producing and reproducing that institutional order—via their compliance with law, taxes, and political, social, cultural, economic activity—a fair return that can be captured by principles that treat the 90% as a collective asset. That fair return can be captured, in principle, by something like the UBI.

But if this is, in fact, a better interpretation of the grounds of a UBI, and it is also true that we need to take into account the nature and domain of the institutional order in question in deriving principles for it, then we need to allow for more variation. We should not believe that there is only one set of principles of justice that applies above some threshold (as in coercion‐based views like Nagel's and Van Parijs's), but not below it. Rather, we ought to think of the correct principles as varying along with the nature and domain of the institutional orders that together secure background conditions for our flourishing. What reciprocity in the provision of collective goods requires at the state level may then be very different from what it requires at the EU level and different again from what it requires at the global level. This would be because the collective goods—and so the “institutional inheritance”—secured at different levels vary in kind, range, and scope, and so the fair return owed would vary as well. It may be, for example, that a smaller UBI is justified (if it is justified) at the EU level—in virtue of the more mediated nature of the collective goods provided at that level—than at the state level. I return to this possibility below.

Notice that on this modified picture, it is no longer true that egalitarian principles mandate a global UBI at the maximum sustainable level. It is no longer true, that is, that a UBI at the EU level is a less‐than‐fully just, nonideal instrument for reaching what egalitarianism really requires. Rather, the EU would be an independent arena of justice, whose principles would need to be sensitive to the particular way in which the EU transmits and reproduces the institutional inheritance to the next generation.

## 4 THE EURO‐DIVIDEND AS A POLICY

So far, I have argued that Van Parijs's coercion‐based justification of the euro‐dividend is unsuccessful and suggested that an alternative view—one that emphasized our mutual contribution to the collective goods necessary to transmit the institutional inheritance from which we all benefit—would do better. In this section, I evaluate the euro‐dividend as a policy and assess whether an alternative reinsurance scheme could both (a) avoid the pitfalls of the euro‐dividend and (b) better match the reciprocity‐based rationale introduced at the end of the previous section.

Van Parijs's euro‐dividend, pitched initially at €200 (adjusted for cost of living, PPS) per person per month (equivalent to 7.7% of EU GDP), is an ambitious proposal. It promises, in particular, four outcomes designed to overcome what Van Parijs calls “Hayek's trap.” Hayek's trap, in brief, refers to the downward pressure on levels of redistribution that happens as a result of the creation of a common market in goods, capital, services, and people. The idea is that once borders are open, tax and social competition will make it increasingly difficult for member states to generate enough revenues to sustain transfers. Such competition could be stymied if redistribution (via the euro‐dividend) were shifted to the EU level, where tax and social competition weigh less heavily. Van Parijs also claims that the euro‐dividend would shield domestic redistributive schemes from competition by partially substituting domestic provision, and so alleviating pressure on member states to fund such provision alone. Finally, the euro‐dividend would support generous cross‐national redistribution. As Table 1 indicates, it would generate, all else equal,^11^ transfers of about 1.1% of GDP per member state in the EU North to fund inflows of about 3% per member state in the EU South and East (calculated assuming each member state must contribute 7.7% of its GDP to fund the PPS‐adjusted euro‐dividend). The euro‐dividend, Van Parijs writes, “foster[s] the pursuit of egalitarian justice both directly, through EU‐level net transfers better protected against social and tax competition than country‐level redistribution, and indirectly, by buffering national‐level redistribution against such social and tax competition.” (Van Parijs, 2019, p. 27)

**TABLE 1:** Net transfers with PPS‐adjusted euro‐dividend^a^

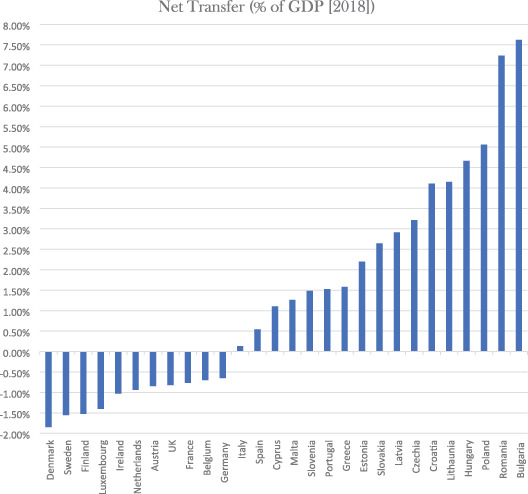

a *GDP and population figures (Eurostat)*. Author's own calculations (percent transfer calculated as the difference between what it would have cost to fund a €200 PPS‐adjusted UBI nationally and what it would cost under the euro‐dividend, assuming each member state contributes 7.7% of GDP via a domestic VAT levy [ca. 19%]).

Second, it promises to increase the stabilization capacity of the EU (Van Parijs & Vanderborght, 2017, p. 232). Whenever a subunit in a federal state undergoes an exogenous shock, one of the ways in which the shock can be alleviated is via federal fiscal transfers. If unemployment in the region goes up, for example, and if—as is mostly the case in federal states—unemployment insurance is organized at the federal level, then money will flow into the region, thus smoothing the income and consumption of those most affected. The EU, however, currently lacks much in the way of such automatic stabilizers, which undermined its ability to react effectively to the differential impact of the financial crisis on Northern and Southern states. The euro‐dividend, given its transnationally redistributive character, would serve to enhance the stabilization capacity of the EU: were a region, for example, to suffer an exogenous shock, the income of many of its inhabitants will decrease; the net transfers required to maintain the euro‐dividend in that country would therefore increase, making up a portion of the loss. (I return to this point below.)

Third, the euro‐dividend promises to avoid the intrusive and expensive bureaucracy that would be attached to mean‐tested benefits (which would also require harmonizing the targets and cutoffs across member states). It would also help to preserve the internal linguistic and institutional diversity of Europe. According to Van Parijs, “The member states' ‘separate forms and traditions of social policy’ have been shaped and should continue to be shaped by largely separate debates. If only because of the linguistic distinctiveness of these debates, a particularly strong version of the subsidiarity principle should apply. In matters of social policy as in many others, it lastingly justifies a degree of decentralization significantly higher than what would be optimal with a mono‐national population of equal size.” (Van Parijs, 2019, p. 27). The euro‐dividend, therefore, would preserve rather than undermine, Van Parijs claims, the self‐determination of member states.

Fourth, the euro‐dividend will reduce pressures on the welfare state from what Van Parijs calls “selective migration” by giving potentially mobile citizens from poorer countries an incentive to stay.

What I will argue is that there are, however, two significant weaknesses that make a euro‐dividend unattractive when compared with alternatives.

First, a euro‐dividend, even pitched at such a low level, is expensive. It would require member states to find fiscal space equivalent to 7.7% of EU GDP.^12^ Van Parijs and Vanderborght's proposal for how to fund it requires an increase of up to 19% VAT (calculated using a VAT basis of about 40% of member states' GDP). To give an idea of how massive that increase would be, consider that funding the *entire* amount of public healthcare spending in Italy (one of the most expensive in Europe) took up 6.6% of GDP in 2018.^13^ The *entire* government spending for Italy's education, defense, *and* economic affairs comes to just over 9%; 7.7% of GDP is equivalent to slightly less than *one‐third* of all Italian social spending. My point is not that such a reform is infeasible. Rather, the point is that, even if it were to be enacted, it would, first, have a very large impact on patterns of consumption and spending (and, indeed, on VAT evasion, especially for services). It is very likely, as a result, that governments would have to either widen the VAT base or increase rates further to maintain the revenues required to fund the euro‐dividend. But even if we leave such dynamic effects to the side, a euro‐dividend would, second, leave states with limited fiscal space, given the need to create room for the additional expenditure.^14^ This, in effect, means that even a small euro‐dividend would severely limit the policy options of all member states. A likely outcome would be disinvestment from public services from education to healthcare to local community services, which are already under pressure in all member states (this is one of the main reasons UBIs are also popular with right‐wing libertarians and silicon valley entrepreneurs). And, once the necessary tax and spending authority has been shifted to the EU level, it would effectively bind all member states for the indefinite future. This would be a direct result of a “joint decision trap”: Once implemented, any proposed legislation to undo it could be vetoed by a blocking minority (Genschel, 2016; Scharpf, 1988). One might think: “Great. This is a positive outcome. Now all member states would be effectively locked into a universal basic income scheme.” Maybe this is a positive outcome, but it would effectively mean that states will have lost a crucial area of self‐determination. A euro‐dividend, in other words, would *undermine* rather than *promote* the institutional diversity realized at the member state level. Van Parijs's claim that it would preserve self‐determination therefore looks shaky. Given the likely impact on domestic fiscal policy, domestic redistributive schemes would therefore be *replaced* (but only partially) by the EU‐level scheme rather than *shielded* from the pressures of competition.

One reply might point to the fact that there is plenty of fiscal space for maneuver, once we factor in the ability of states to make savings on the bottom part of welfare entitlements. States could, that is, cut back on welfare entitlements where this will be compensated by the euro‐dividend, while preserving a “floor” beneath which no one should fall. There are two main problems with this reasoning. First, it is unlikely, given how small the euro‐dividend is initially pitched, that this could save much. One optimistic estimate would be that this would lower the cost, on average, by 20%; this would still mean extracting an extra 6.4% of GDP (with a 15.2% VAT increase) at the European level, which is still equivalent to what is required to fund the entire public health service in Italy. More importantly, what the possibility of such a saving shows is that there is a tradeoff with the “real freedom” rationale that makes a UBI attractive in the first place. The more other entitlements are simply replaced by the new UBI, the less “real freedom,” the UBI will provide. Indeed, if one of the main arguments in favor of a UBI is that it would increase the bargaining power of workers by providing a credible opt‐out, then €200 in PPS a month would not provide enough to say “no” (note that this would be true even if states were to avoid cutting other entitlements). Furthermore, the low level would vitiate the other important strength of a UBI—namely that it would serve to eliminate the “unemployment trap” created by standard minimum income entitlements with high clawback rates (i.e., the rate at which benefits are reduced as income increases).^15^ Given that the €200 a month UBI will need to be “topped” up by standard entitlements, clawback rates, all else equal, will remain high, and so the unemployment trap would remain.^16^ The third main problem is that replacing current entitlements with the UBI will inevitably create some winners, but also some losers. Most estimates for lower‐level UBIs conclude that, for example, single, unemployed, childless workers would fare worse, whereas families would fare much better (Hirsch, 2015; Immervoll, 2017).

The second weakness of the euro‐dividend is that its capacity to act as a stabilizer in the event of exogenous shocks is limited compared with other, much less expensive alternatives. If we assume a 5% GDP shock to the economies of Italy, Spain, Portugal, Greece, and Cyprus, there would be a net transfer, all else equal, into those economies of, on average, 0.38% of their GDP compared to what they were receiving before the shock. To fund the transfer, other EU countries' contributions would need to increase, on average, by about 0.08% of their GDP (along with the total extraction, which would now require 7.8% of EU GDP). This shift would reflect the fact that the countries receiving the shock would now be relatively poorer compared to the others, and so less able to fund their part of the euro‐dividend via VAT.^17^ Therefore, with an overall extraction of 7.8% of GDP (funded via VAT), the euro‐dividend would net only a 0.38% adjustment in a severe downturn.

To compare, let us consider a policy that is specifically designed as an inter‐state insurance mechanism that pays out in significant downturns. Take, for example, various proposals for EU‐level unemployment reinsurance schemes, which pay out whenever there is a significant year‐on‐year increase in unemployment in member states. See, for example, schemes suggested by (Arnold, Barkbu, Ture, Wang, & Yao, 2018; Carnot, Kizior, & Mourre, 2017; Dolls, 2019; Dullien, 2014). For purposes of comparison, let us single out a proposal that is, I believe, the most promising. While I will not be able to give a full defense, I will seek to show that it would not only serve as a more significant stabilizer but also meet, much less expensively, all of the claimed advantages of the euro‐dividend. The scheme is organized in the following way. See (Arnold et al., 2018) for details and simulations. Every year member states pay 0.35% of GDP to finance a reinsurance fund.^18^ For every 1% increase in the unemployment rate over the 7‐year moving average in total unemployment, the member state receives a payout of 0.5% of their GDP.^19^ So, for example, if the scheme had been in place in 2013, Greece would have received a net payout equal to 5.65% of its GDP (given that its unemployment rate was 12% higher than its 7‐year moving average in 2013). Cumulatively, in the period between 2009 and 2016, Greece would have received net transfers of about 20% of GDP (also Spain between 2008 and 2015) (Arnold et al., 2018, p. 14). With the euro‐dividend, net transfers into Greece would have increased by 0.11% in 2012–2013, and cumulatively, between 2009 and 2016, it would have received 3.35% of GDP. That figure is six times smaller than the reinsurance proposal I am proposing; the euro‐dividend's capacity to stabilize in a downturn is therefore much weaker.^20^

The proposal has a number of further advantages. First, it is much less expensive than the euro‐dividend. Whereas a euro‐dividend of €200 per person requires an additional 7.7% extraction in EU GDP, the reinsurance proposal only requires 0.35%. For this reason, it does not crowd out the fiscal space of member states, or otherwise encroach on their capacity to tax, and so would have a much smaller effect on self‐determination. Second, it does not require any intrusive bureaucracy or means‐testing, given that the funds are disbursed without conditionality. The funds, furthermore, are disbursed automatically, and so there is no need for extensive supranational negotiation (compare the operation, for example, of the European Stability Mechanism or the planned post‐COVID recovery funds). Second, the unemployment statistics produced by Eurostat for each member state are produced using the same methodology; there is, as a result, little scope for gaming the system. They are also a very reliable indicator of an external shock—much more reliable than real GDP statistics.^21^ Third, the funds, given their large stabilizing character, will have a much greater effect on maintaining incomes in a downturn than the euro‐dividend. This will make emigration less likely, and so reduce pressure from migration on host states.

What about the effects on tax competition? First, there is reason to question whether the euro‐dividend would in fact alleviate tax competition in the first place. Let us assume that, optimistically, the euro‐dividend would only replace the bottom part of welfare spending, and that expenditure on public goods and services at the domestic level would stay the same. Given that governments would still need to top up the €200 basic income in order to provide a living wage, they would still have incentives—assuming such incentives actually exist and do have an impact on government policy—to attract capital and investment by reducing welfare entitlements up until the €200 base level. And, second, if we assume that raising the revenue to fund the euro‐dividend would put pressure on all other sources of revenue (as I have argued above), it is likely that those taxes that are most likely to be affected by competition, such as corporation tax, would face even more intense downward pressure than would have otherwise been the case.

A reinsurance fund, on the other hand, would likely fare better. If we assume that there will be most pressure on member states to scale down their welfare systems in an attempt to attract capital and labor during a significant downturn, and we assume that pressures to impose austerity increase in such a downturn (especially if states are already indebted), then a reinsurance fund, as we have seen, does better in stabilizing and smoothing incomes, and so would also do better in reducing such downward pressures. Because, that is, a reinsurance fund generates large net inflows precisely when pressures on the state's revenue‐generating capacity are greatest, it will also be more effective in alleviating that pressure when it is needed most.

I now want to argue that the reinsurance scheme would also be preferable according to the reciprocity‐based conception of EU justice introduced at the end of Section 3. Note that the reinsurance fund would not have the ongoing and significant redistributive effect of the euro‐dividend (recall Table 1). Indeed, all of the most important defenses of such a reinsurance fund hold that it should be designed to be *ex ante* distributively neutral, which means that no member state, over the longer run, will be either a net beneficiary or net contributor (by, for example, adding provisions that require states that draw more down on the fund than other states to pay back more in the future [so‐called “clawback” provisions or provisions for “experience rating”]).^22^ But ought we to accept such *ex ante* distributive neutrality (often defended on the basis of actuarial fairness—i.e., states that face higher risks should pay more)? I now want to argue that, on the best understanding of inter‐state EU justice, we ought to reject *ex ante* distributive neutrality when designing the reinsurance fund. The best understanding of what justice requires for the EU, that is, ends in the conclusion that we ought to conceive of the EU as an insurance union, but one that redistributes from richer to poorer member states even in the longer run. It therefore retains some, but not all, of the redistributive effects of the euro‐dividend, but on very different normative grounds.

Recall that, on that reciprocity‐based view of justice I alluded to earlier, we owe much more stringent obligations of distributive justice at the member state than at the EU level. This is because (a) the collective goods secured at the EU level are less comprehensive than those provided at the domestic level, and (b) the EU is ultimately parasitic on its member states for the transmission and provision of those goods. This is not to say that no redistribution is required at the EU level, but the principles governing such redistribution ought to be different precisely because cooperation at that level differs in both degree and kind.

If we take *member states* rather than *individuals* as the primary distributive unit at the EU level, then what principles of redistribution would apply at that level? Given the narrower focus of this article, we can specify our question further: What principles should govern member states with respect to cooperation within the EU, that is, given the benefits and costs that are foreseeable within the union? Elsewhere, I have argued that the principles governing such cooperation should be grounded, as at the domestic level, in an account of fair reciprocity (Sangiovanni, 2013). More specifically, what member states owe one another is a fair return specified by how each member state would have insured against, in our case, the risk of an exogenous shock, had they known the risk profiles of each state (the degree of exposure to an exogenous shock, given background circumstances such as level of indebtedness, quality of governance, and efficiency of labor institutions) but not which member state they are.^23^ From behind such a modified veil of ignorance, states would choose insurance that reflects an average level of exposure to risk. The insurance policy chosen will therefore not be as generous in its coverage and contribution rates as if it had been chosen by a high‐risk state, but more generous than if it had been chosen by a low‐risk state. Designing the insurance pact in this way makes it the case that there will be redistribution from low‐ to high‐risk states *even in the longer run*, and hence explains why we should reject *ex ante* distributive neutrality when designing an EU reinsurance fund.

Indeed, we can go further: Once we ask what insurance policies would be chosen had member states known the distribution of risks but not their place in the distribution, we should also accept that contribution rates (i.e., premia) should vary as well. We should expect, that is, richer states—who have more leeway to adjust in downturns—to pay more into the fund relative to poorer states—for whom the opportunity costs of adjustment in a downturn will be, on average, larger. This follows from the fact that, were MSS not to know whether they have a high‐ or low‐risk profile vis‐à‐vis exogenous shocks, but did know both the relative distribution of risk profiles among MSS and the opportunity costs faced by each state in adjusting to such costs, they would choose to insure such as to equalize the opportunity costs of adjustment across MSS. As a result, states that have lower costs of adjustment (who will also be most likely to be the states with low‐risk profiles) subsidize states with higher costs of adjustment (and higher risk profiles). One way to implement such variation in rates would be, for example, to weight the contribution rate according to the deviation of each member state from the EU GDP per capita average, such that richer states pay in more each year than poorer states. In downturns, this would mean that poorer states receive relatively more than richer states, and in upturns, they are allowed to retain more of the surplus (relative to their GDP). While redistribution across member states would not be as extensive as under a euro‐dividend, it would still be significant (and significantly more than is envisioned by extant proposals for reinsurance). Note that it may even be the case, depending on the distribution of risks and adjustment costs (i.e., if most states are high risk and have high costs of adjustment), that low‐risk states end up, even over the long run, being net *ex ante* contributors to the scheme (i.e., that they would be better off without any insurance scheme in the first place than with the scheme as proposed).

The logic of redistribution here is analogous to compulsory domestic social insurance (e.g., unemployment insurance). The risk that any person becomes unemployed over their life varies according to such factors as level of education, talent, skill, gender, and sector. We do not, however, expect individuals who are at high risk of unemployment to pay more for their unemployment insurance than those who are at lower risk, or for the level of coverage to reflect the most the market will bear (with imperfect information and adverse selection, this is likely to be lower than we would optimally want). This, we believe, is unfair, especially since many of the most important factors that explain individuals' risk of unemployment are “arbitrary from a moral point of view,” that is, those who face higher risks do not *deserve*, as a result of those factors, to do worse than others who face lower risks. One way to model our judgments is to ask what kind of unemployment insurance individuals would have chosen had they known the distribution of risks but not their place in that distribution, and hence not known what antecedent risk they have of becoming unemployed.^24^ Taking this route implies that there will be redistribution from the low risk to the high risk, and we design both contribution and coverage rates to reflect that fact. The normative rationale is equivalent, mutatis mutandis, for member states at the EU level.

## 5 CONCLUSION

In this article, I have sought to continue the discussion that Van Parijs and Rawls began many years ago. I have argued that Van Parijs's arguments for a UBI pitched at the EU level—a euro‐dividend—do not support such an extension for both philosophical and policy‐based reasons. I suggested that principles of justice for the EU grounded in reciprocity could do better and can provide the rationale for an unemployment reinsurance fund designed to indemnify states in severe downturns.

## Endnotes

1 This remains true, although a growing number of younger philosophers are beginning to address it more systematically.

2 The passage is drawn, *verbatim*, from the *Law of Peoples*, p. 43n.

3 This, of course, continues to be true.

4 The euro‐dividend is defended at much greater length in Van Parijs & Vanderborght (2017).

5 He also refers to “scaling up” as a demand of equal concern and respect, but did not elaborate the claim.

6 See also (Van Parijs, 2007), which makes a similar argument. To my mind, the argument at this point has more in common with Blake (2001 than Nagel (2005), but nothing turns on that here.

7 I challenge Nagel's stronger formulation in Sangiovanni (2012) and Sangiovanni (2011).

8 This would bring Van Parijs's argument even closer to Blake (2001).

9 We could go even further: Suppose that the poor in the post‐attack society could emigrate to other states in which they would receive no worse treatment than they would get under the rich group's proposed reforms, and assume that the poor would rather not do so. Would that possibility render the rich group's claim to undo the egalitarianism of the current system any more plausible? Could the rich group plausibly claim to the poor, after they had enacted their anti‐egalitarian reforms: “love it or leave it”? Or: “If you decide to stay here, you now do so voluntarily (given the possibility of emigrating), and so we no longer have any obligations to you to maintain an egalitarian scheme”? I say more about cases like this in Sangiovanni (2012), including what I call the “spurious role of consent”.

10 I make this point at greater length in Sangiovanni (2012).

11 The estimates assume that the economies otherwise remain the same. For our purposes, we do not need a more dynamic model, which would bring in controversial assumptions, among other things, regarding labor supply post‐reform.

12 Calculated using 2018 figures.

13 OECD.

14 I leave aside the effects such an increase in VAT would have on household expenditure and VAT evasion, which are difficult to model. Very important in such a calculation would also be whether the VAT base would be widened or narrowed. Consider that while well over half the population would benefit to some extent from the euro‐dividend after taxes, goods and services would be much more expensive at point of sale, which we can expect to have a large effect on spending patterns. Also consider that households in the middle quintiles of the income distribution would obviously benefit less than those at the lower end (because they spend more both absolutely and relatively on purchases subject to VAT). Those in the upper quintiles would be overall net contributors (unless they massively divert spending—for example, by spending abroad—or otherwise evade VAT).

15 This point is also made by (Vandenbroucke, 2017).

16 One might argue that these figures are misleading because we are only considering the revenue side of the euro‐dividend. If we consider that, after all, the amounts raised via VAT are then returned directly to citizens, the *net* contribution of each citizen will be much smaller. Even if we leave aside the likely effects of a euro‐dividend on labor supply and demand, prices, and so on, netting out what each income quintile would likely pay to fund the euro‐dividend from the amount they receive back as a basic income confirms that the amount left over to reduce conventional welfare entitlements would, in most cases, be very small (except for poorer member states). It also confirms that for, many the cost of a euro‐dividend remains high. For example, in Denmark, on average, the top three income quintiles (i.e., 60% of the population) would need to pay an additional €2000 each year, net, to fund the euro‐dividend. Denmark would then in effect send 1.85% of their GDP from this levy abroad (see Table 1 above), and use the rest to pay a modest subsidy of about €47 (€38 PPS) per month to the lower two income quintiles. But €47 per month per capita will not be enough to replace much in the way of social spending. A similar pattern is repeated for all of the contributing states. A country like Romania, on the other hand, would be a net beneficiary across all income quintiles; indeed, they would have a net inflow of about 7% of their GDP yearly (see Table 1 above), which would fund a net income of, on average, an additional €63 (€97 PPS) per person per month for every Romanian citizen. Given the much lower levels of social spending in Romania compared to the EU28 average, these amounts would be significant. Countries like Slovenia would land somewhere in the middle, such that the top two income quintiles would see, on average, an additional €325 (€537 PPS) on their yearly tax bill (again net of what they get back from the euro‐dividend), which would contribute to funding a €64 (€84 PPS) per month per capita basic income for the bottom three income quintiles (the remainder would be funded by the 1.49% of their GDP they receive from richer member states [see Table 1 above]). [Own calculations available on request, using VAT receipts from each income quintile for each country, and assuming, unrealistically, that the proportions remain the same before and after the extra VAT imposition.]

17 Calculated using the same figures, adjusted, as Table 1.

18 As envisaged by Arnold et al. the fund can borrow on capital markets if it is in deficit. This allows for a greater degree of inter‐temporal smoothing, and helps in those cases where insurance is most needed (i.e., where shocks have been frequent and severe).

19 The 7‐year moving average is to ensure that the fund only tracks shocks, rather than rises or falls in long‐term, structural unemployment. The unemployment rate deviation from the 7‐year average is intended as a proxy for a shock.

20 Of course, Greece would have been receiving consistently about 3.65% of its GDP as a transfer under the euro‐dividend, but the point is that that figure is not very responsive to year‐on‐year shifts in GDP, which is all that matters for its effect as a stabilizer.

21 There are three reasons that unemployment statistics are a better indicator than real GDP. First, ex‐post revisions to unemployment statistics are smaller than ex‐post revisions to real GDP. Real GDP is also much more volatile over shorter time‐spans, and so is less reliable as an automatic trigger. And finally, according to Arnold et al., “if real GDP is measured as a deviation from its moving average, it is less countercyclical than the unemployment gap, because growth rates start picking up after the trough of a cycle, well before the unemployment gap is closed” (Arnold et al., 2018, p. 15). There is also the problem of countries like Ireland and Luxembourg, whose GDP statistics are distorted by the presence of multi‐national corporations relocating there. Is the value added by these relocations really a reflection of economic activity in Ireland? Consider that real GDP growth in Ireland increased by 26.3% (!!!) in 2015.

22 This is exactly what (Arnold et al., 2018), for example, recommend along with nearly everyone else who has advocated such a fund. See, for example, (Beblavý, Gros, & Ilaria, 2015; Bénassy‐Quéré et al., 2018; Carnot et al., 2017; Dolls, 2019; Dullien, 2017).

23 I discuss such an insurance rationale at greater length in Sangiovanni (2013).

24 Cf. (Dworkin, 2000), Ch. 2.
